# Promoting Precision Cancer Medicine through a Community-Driven Knowledgebase

**DOI:** 10.3390/jpm4040475

**Published:** 2014-12-15

**Authors:** Nophar Geifman, Izhak Haviv, Razelle Kurzrock, Eitan Rubin

**Affiliations:** 1 Shraga Segal Department of Microbiology, Immunology and Genetics, Faculty of Health Sciences, Ben Gurion University, Beersheva 8410501, Israel; E-Mail: erubin@bgu.ac.il; 2 National Institute of Biotechnology in Negev, Ben Gurion University, Beersheva 8410501, Israel; 3 Faculty of Medicine in the Galilee, Bar Ilan University, Ramat Gan 52900, Israel; E-Mail: izhak.haviv@biu.ac.il; 4 Center for Personalized Cancer Therapy and Division of Hematology and Oncology, Moores Cancer Center, UC San Diego, La Jolla, CA 92093, USA; E-Mail: rkurzrock@ucsd.edu

**Keywords:** cancer, case reports, genetics, knowledgebase, personalized medicine, precision medicine

## Abstract

Increasing efforts are being dedicated towards improving cancer care via personalized medicine. These efforts depend to a large degree on the availability of a knowledge foundation. Unfortunately, existing knowledge linking cancer drugs and potential efficacy biomarkers is in its infancy; and where links are known, they are frequently unstructured and poorly documented. We have developed a new open-access knowledgebase for precision cancer medicine (the PCM Wiki and Knowledgebase). This knowledgebase was constructed using an innovative, two-pronged approach involving a structured knowledgebase at the back-end, and an intuitive knowledge-sharing interface and user-friendly query engine in front. The knowledgebase was seeded with several patient case reports and information was mined via text-mining and literature review by human curators. Using our novel Wiki-based platform to present and share knowledge stored in the PCM knowledgebase, users are able to suggest corrections, propose additions or point to errors in the knowledgebase. The result is a community-driven evolving knowledgebase holding integrated and consolidated knowledge of markers and indications for personalized cancer medicine. We suggest that the PCM Knowledgebase and Wiki could serve as an important tool for the advancement of clinical trials and care in the field of precision cancer medicine.

## 1. Introduction

Personalized, or precision medicine is the tailoring of specific therapeutics for a specific individual, utilizing pharmacogenetic and/or pharmacogenomic information [1,2]. This paradigm is likely to become the future of medicine, largely due to significant advancements in our ability to measure and capture genetic, genomic, phenotypic and other -omics information [2,3]. Though much progress has been made towards realizing the goal of personalized medicine, the translation of such information into clinical care still requires the development of resources capable of capturing, integrating and analyzing the multiple information sources in conjunction with other clinical information [4,5]. Indeed, increasing efforts are being dedicated towards improving cancer care via personalized medicine. These endeavors are largely based on comparing the properties of a given tumor to prior knowledge about drug efficacy. In other words, determining which molecular states are associated with the efficacy of which drugs has become a major driving force in personalized cancer medicine.

Precision cancer medicine (PCM) can be considered as the exemplar for personalized medicine. Due to the special properties of cancer and challenges in choosing therapeutic approaches, considerable efforts have been dedicated to using focused genetic screening, coupled with prior knowledge (mostly about drugs targets), to propose efficacious drugs. In other efforts, such as in the WIN-sponsored WINTHER clinical trial [6], over- or under-expressed transcripts are considered for their value in predicting drug efficacy. In this approach, tumor-specific gene expression patterns are compared to prior literature-based knowledge, using known or inferred links between drug efficacy and alterations in gene expression. Both approaches, like similar strategies, require comparison of the properties of the tumor to prior knowledge.

In light of the importance of prior knowledge for PCM, it is unfortunate that most existing knowledge linking cancer drugs to potential biomarkers is unstructured and disorganized. Moreover, even in those cases where specific drug-biomarker links are sufficiently characterized to become standard of care (such as BRAF:V600E-Vemurafenib or ALK-Crizotinib), such links are currently restricted to one or only few cancer types (e.g., melanoma or lung cancer, respectively). The effect on other cancer types where a biomarker is rarer is often not even anecdotally described. While such scarcity of structured knowledge about drug-marker links is gradually being rectified by multiple clinical trials conducted world-wide (such as the WINTHER project), the rare prevalence of the potential new indications could severely delay the dissemination of any knowledge obtained.

Other potential sources of information addressing marker-drug links include cancer genomics and structured marker databases. Genomic cancer data is rapidly accumulating in mutations databases, such as ICGC [7], COSMIC [8], TARGET [9] and TCGA [10]. However, these databases contain little if any information about specific drug responses and/or follow-up information. Structured knowledgebases containing information about drug targets and mechanisms of action are also available [3,4], and can be used in many cases to define efficacy-predicting mutations, although such knowledgebases are all developed around the strong yet simple drug-gene associations that dominate the scientific literature. Furthermore, knowledge linking genes, their expression levels, cancer types and drug efficacy is less well documented, especially for drugs for which cancer genome involvement in the pharmaco-dynamic mechanism is less transparent, such as Avastin, Perifosin, or Regorafenib.

One of the knolwedgebases enriched in information about drug-gene interactions is the Comparative Toxicology Database (CTD) [11]. This knowledgebase, which was used for the WINTHER trial, is one of the most comprehensive resources of expression-drug interactions available. With stringent filtration (e.g., removing contradicting evidence), it can be used for personalized cancer medicine research. However, it is too partial, missing above all information about experimental drugs. Moreover, since it was not specifically aimed to assist in personalized cancer medicine, CTD contains much information on non-drug substances and other irrelevant information. As a result, its use in the context of clinical decision-making requires massive filtration and processing efforts, along with additional exhaustive literature review. Although other commercial databases have also been developed for the same purpose, these too are rarely geared to support personalized cancer medical care. The Integrity database [12], for example, contains a large amount of marker-related information, but was specifically designed to support research rather than for clinical care. Another related database, the My Cancer Genome database [13], provides users with information regarding cancer-associated genes, possible targeted therapies and related clinical trials. Still, cancer mutation-related information in this database is currently limited to only a handful of cancer types. Moreover, none of the existing knowledgebases offer an intuitive interface that allows end users to share their knowledge, and no knowledgebase in this domain allows users to contribute their own knowledge freely, immediately and transparently.

On a more fundamental level, high throughput datasets have accepted standards for authors reporting their results so that the utility of any experimental empirical data can be maximized. The main contributor of these standards is the Functional Genomics Data Society (FGED) [14]. Currently, however, there is no project within FGED that addresses clinical information about cancer patient responses as guided by biomarkers. Furthermore, even where genotype-phenotype databases do exist with such an aim, such as the personal genome project [15], the approach to the data is more appropriate for diploid genomes and is not well adapted to cancer genomic data (e.g., denoting the percentage of reads indicating the variant on which the drug was chosen). Thus, the need for standardizing data deposition and precision cancer case reporting is urgent.

With this in mind we have developed a new open-access knowledgebase for precision cancer medicine. This knowledgebase was developed using an innovative two-pronged approach involving a structured knowledgebase at the back-end and an intuitive knowledge-sharing interface in front. Knowledge is represented using formal knowledge representation methods to ensure it can be mined, merged with other data sources, searched and filtered, making full use of state-of-the-art computer science methods. The structured knowledgebase can also be exploited to make informed decisions in clinical trials or in practice. In addition, we have created an intuitive interface that allows the user community to record their knowledge by correcting or adding information to the knowledgebase. The result is a knowledgebase that contains integrated and consolidated knowledge of genetic markers, indications and treatments in personalized cancer medicine. To demonstrate the usefulness of this approach, the knowledgebase was seeded with actual case reports and information mined from the relevant literature that can now be edited by the community.

## 2. Results and Discussion

### 2.1. The Precision Cancer Medicine Knowledgebase and Wiki

**Figure 1:**
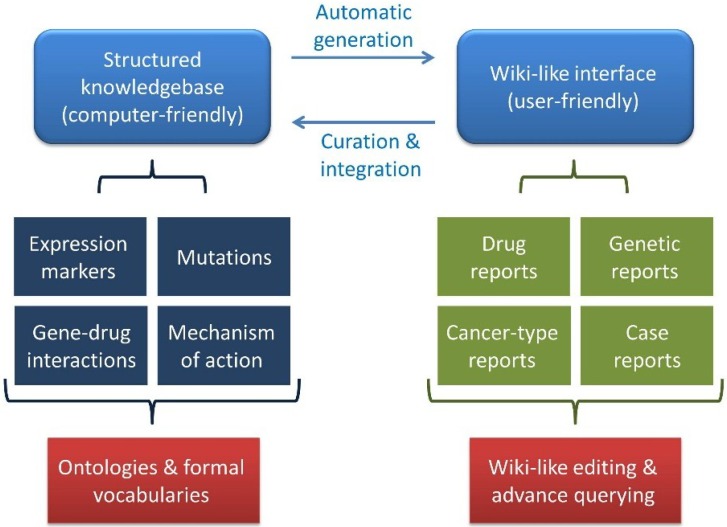
The Precision Cancer Medicine Wiki and Knowledgebase model. The precision cancer medicine (PCM) Wiki and Knowledgebase (KB) model comprises a structured knowledgebase together with a user-friendly Wiki-like interface.

We have developed a unique knowledgebase utilizing a novel approach for capturing, storing and sharing knowledge concerned with precision cancer medicine (http://pcm-wiki.bgu.ac.il). The knowledgebase is comprised of two arms, a structured knowledgebase on the one hand and a user-friendly Wiki-like interface on the other (Figure 1). The knowledgebase holds information mined from the literature regarding different types of cancers, drugs, genetic markers and their co-occurrences in publications, as well as case studies introduced through a specialized form. This information is represented, wherever possible, by appropriate ontologies and formal vocabularies that allow a “computer-friendly” representation of the data. To render these data accessible to humans, the knowledge was transformed to the PCM Wiki interface, where the information stored in the knowledgebase is presented on two types of pages, namely information reports summarizing literature-mined information regarding cancer types, drugs and genetic markers and case reports describing actual precision cancer patient reports submitted by the community. The use of the Wiki platform allows users to suggest corrections, offer additions or point to errors in the knowledgebase. Any such suggestions become immediately visible to other users for further revision or rejection. The result is a community-driven knowledgebase holding integrated and consolidated knowledge of markers and indications for personalized cancer medicine.

In addition to the Wiki, users can query the knowledgebase through a query engine designed for the search of specific information or case reports (http://pcm-crs.med.ad.bgu.ac.il/Query/). This query engine is designed to allow physicians to find “patients like mine”. Using such a query engine allows the clinician to search for evidence that a particular treatment would benefit a patient by searching for patients who shared similar characteristics as the patient at hand in situations were no clear guidelines are available. A case report of a patient with good outcome could help prioritize possible treatments. Indeed, a similar approach is already in use in a web tool called “Patients like me” that allows users to find and compare their disease history and treatment course to other patients sharing similar conditions or symptoms [16]. The tool we propose here, however, is specific for cancer precision medicine and is aimed for use by physicians rather than patients.

To simplify the contribution of case reports from our community of users, we have developed a simple user-friendly case report submission form (http://pcm-crs.med.ad.bgu.ac.il/form.html). Users provide all of the information pertaining to a case (such as diagnosis, molecular aberration, personalized treatment, treatment outcomes, *etc.*). This data is subsequently used to automatically generate a case report page on the PCM Wiki.

The submission mechanism sets a uniform structure for the initial submission, thus keeping case report Wiki pages consistent. In addition, the use of a standard web form means that users do not have to familiarize themselves with Wiki page editing. On the other hand, given that a standard form may not be suitable for all possible cases, the use of a Wiki allows for the necessary flexibility in capturing knowledge. Since variability in precision cancer medicine cases is high, it may be the case that the form does not contain all of the fields needed to properly capture a given patient report. In such cases, the user can add any additional fields or patient data through editing of the Wiki page generated by the standard form. Indeed, in one of the first case reports that we attempted to contribute through the form (provided by Prof. Shmuel Ariad, Soroka University Medical Center, Beersheva, Israel), the information could not be completely captured by the form alone as it involved reversals of diagnosis (from BRCA1 negative to positive), a possibility we did not consider likely when designing the form. In this case, the ability to edit the submitted report was essential for our ability to faithfully present the case.

Another advantage of the use of the patient case submission form is that it allows for formal knowledge representation. With this form, users can choose a diagnosis from a provided list of International Classification of Diseases for Oncology (ICD-O) cancer names or from a list of cancer types compiled from the Disease Ontology. By allowing users to select diagnoses from lists of formal terms taken from medical vocabularies and ontologies (*i.e.*, the Disease Ontology and ICD-O), case report data collected by the submission form can be easily formalized, thus making it amendable to complex computational analyses. This formalization of the data stored in the PCM knowledgebase can be easily extended by incorporating appropriate formal terminologies to describe other fields of the form (such as using RxNorm [17] to represent drugs and treatments). Capturing subsequent edits introduced through the Wiki interface is also relatively straightforward. The Wiki stores, all changes introduced to any page by default. Upon mining these page edits, it is possible to correct the information stored in the structured knowledgebase. Although this step is currently conducted manually, it can be automated (at least in part) in the future by using text mining technologies [18,19].

A possible concern with direct contribution of patient information is the potential for compromising patient privacy. In this context, it may be worthwhile to consider the PCM Wiki as a rapid publication journal in that it only includes information that is also included in journal case reports. Contributors merely need to adhere to the ethical guidelines of journal case reporting to ensure that patient privacy will not be compromised. As with any other use of patient information, it is the contributor’s responsibility to adhere to HIPAA or equivalent privacy regulations. Nevertheless, to ensure that private patient information is not introduced accidently or deliberately, cases are manually reviewed periodically. In future, when the number of cases reported becomes too large for manual review, an automatic review mechanism (for example using simple text mining to detect mentions of names, addresses, *etc.*) will be needed.

### 2.2. Mining Drug, Gene and Disease Associations from the Literature

**Figure 2:**
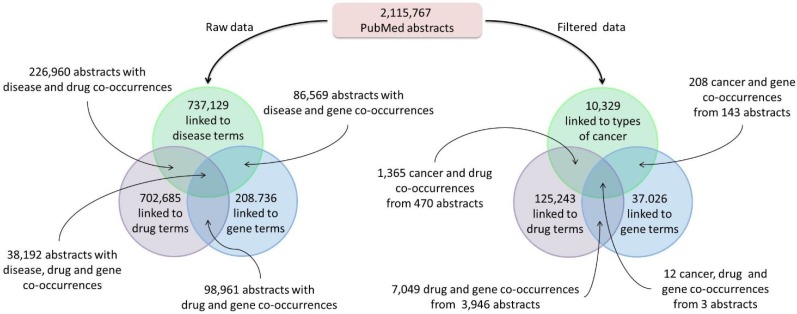
Text mining results. The process of mining Medical Subject Headings (MeSH) terms associated with PubMed abstracts yielded associations with disease, drug and/or gene terms (**left**). The resulting data were filtered (**right**) by selecting only those abstracts linked to cancer and by selecting only abstracts linked to specific types of MeSH categories (See *Clustering Analysis* in the Experimental Section).

In addition to precision cancer medicine patient case reports, the PCM Wiki and Knowledgebase was seeded with information about associations between types of cancer, treatments and genes mined from the literature. Using a simple knowledge extraction pipeline, MeSH terms associated with over two million PubMed abstracts were mined for occurrences of drug, disease and gene terms (Figure 2). From this mining process, we were able to capture the co-occurrence of different diseases, drugs and genes within the biomedical literature. Furthermore, since these associations were mined using terms from formal ontologies and vocabularies, such as the Disease Ontology and RxNorm, advanced representations and mining could be applied to this type information.

Once captured, these associations and co-occurrences were used to create the drug, disease and genetics reports that are presented in the PCM Wiki. For each cancer type, drug or gene, a report summarizing the co-occurrences of this entity with the other entity types was created. For example, for the cancer type “Melanoma”, the report summarizes its co-occurrence with different drugs and genes and includes, for each co-occurrence, a link to the PubMed abstract from which the co-occurrence was captured.

**Figure 3:**
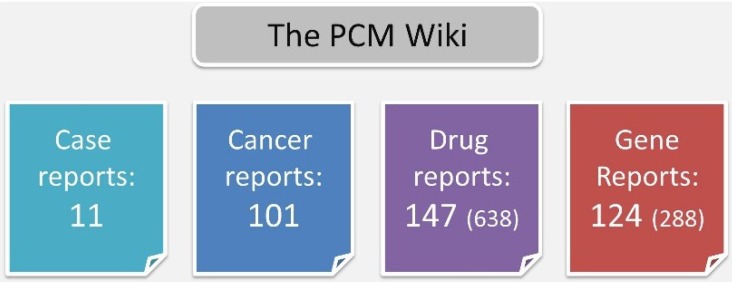
Cancer, drug and gene reports. For each entity (cancer type, drug or gene), a report summarizing its co-occurrence in the literature with other entities was generated. For drugs and genes, only reports with >10 co-occurrences were included. The total number of reports prior to this filtration is given in parenthesis.

The MeSH-based pipeline yielded 101 cancer type reports, 638 drug reports and 288 gene reports. For the Wiki, we chose to include only drugs or genes with >10 co-occurrences (N = 147 and N = 288 for drugs and genes, respectfully) (Figure 3).

**Figure 4:**
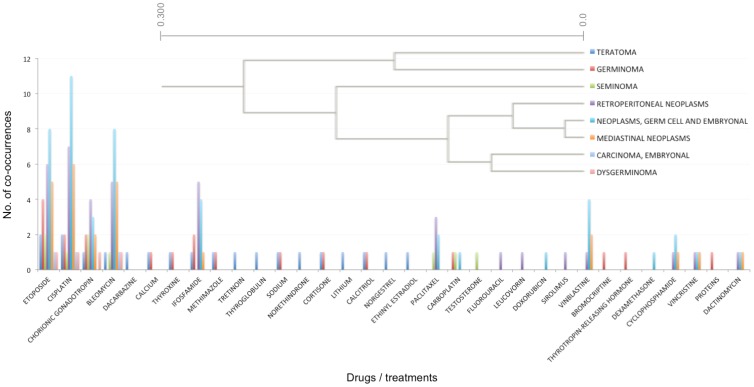
An example cluster generated by hierarchical clustering of cancer types. Cancer types were clustered based on their association (co-occurrence in PubMed abstracts) with different drugs and treatments. The top right corner illustrates the clustering dendrogram, while the plot illustrates the number of co-occurrences for each cancer type and drug/treatment.

**Figure 5:**
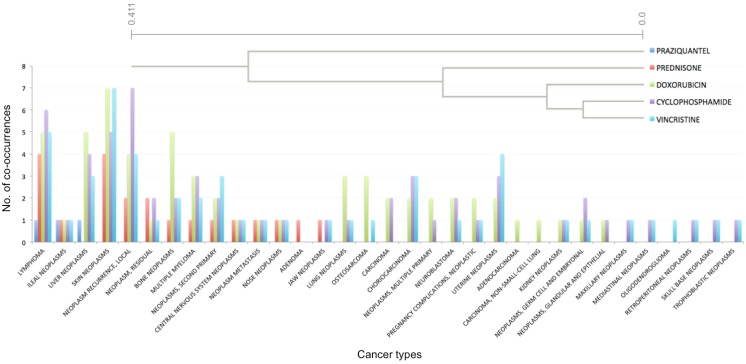
An example cluster generated by hierarchical clustering of drugs and treatments. Various drugs and treatments were clustered based on their association (co-occurrence in PubMed abstracts) with different types of cancer. The top right corner illustrates the clustering dendrogram, while the plot illustrates the number of co-occurrences for each drug/treatment and cancer type.

To explore the usefulness of capturing this type of information, we used these data to cluster cancer types and therapies. Gene-cancer type-drug co-occurrence tables were used to cluster different cancer types based on their co-occurrence with different drugs and therapies (a selected example is presented in Figure 4) and for clustering different drugs and treatments based on their co-occurrence with different types of cancer (a selected example is presented in Figure 5).

Our results indicate that despite the simple mechanism used for obtaining data, the knowledge we have extracted is sufficient and accurate enough to draw conclusions. In one example (Figure 4), different types of cancer were clustered based on their associations with various drugs and treatments. This cluster combined many germ cell and embryonic cancers, such as “Germinoma”, “Dysgerminoma”, “Teratoma”, “Seminoma”, “Neoplasms, germ cell and embryonal” and “Carcinoma, embryonal”. These cancer types were clustered based on their high co-occurrence with treatments, such as: “etoposide”, “cisplatin” and “bleomycin”. Each of these drugs has a different mechanism of action. Etoposide is a topoisomerase inhibitor, cisplatin triggers apoptosis by causing crosslinking of DNA, while bleomycin causes DNA breaks. This cluster of cancer types was also generated due to their association with “Chorionic gonadotropin”, known to be produced by some cancer types [20]. Indeed, in our data, “Chorionic gonadotropin” was associated with “choriocarcinomas” and “trophoblastic neoplasms” for which human chorionic gonadotropin is a marker.

In a second example (Figure 5), different types of drugs/treatments were clustered together based on their associations with various cancer types. Specifically, vincristine clustered with cyclophosphamide, doxorubicin, prednisone and praziquantel (in increasing order of distance). Vincristine is used for treatment of several cancers, including lymphomas, acute lymphoblastic leukemia (ALL), and nephroblastoma. Our pipeline identified abstracts associating vincristine with lymphomas, as well as skin neoplasms, local recurrent neoplasms and uterine cancer. In our knowledgebase, cyclophosphamide was mostly associated with local recurrent neoplasms, lymphomas, skin neoplasms and liver neoplasms. A more complete literature search revealed that cyclophosphamide is most often used for the treatment of lymphomas, some forms of brain cancers, leukemia [21] and some solid tumors [22], in combination with other chemotherapy agents. Doxorubicin is used to treat a wide range of cancers, including hematological malignancies. Prednisone, a synthetic corticosteroid, is used to help treat some leukemias, lymphomas, and other types of cancer (usually along with chemotherapy). These treatments were grouped together due to their high association with several cancer types, including “lymphoma”, “multiple myeloma”, “skin neoplasms” and “neuroblastoma”. In addition to the drugs/treatments mentioned above, the cluster also included praziquantel, a trematodicide used for the treatment of schistosome infections and infections due to liver fluke. In the PCM knowledgebase, praziquantel was linked to cancer through several PubMed abstracts. In one of these, praziquantel was described as successful in treatment of a pseudo-tumor form of hepatic distomatosis [23]. In a second instance, a case of ileocolonic schistosomiasis presenting as lymphoma was described [24].

These two examples illustrate that the cancer type-treatment associations data are concordant with current medical knowledge, as reflected in the grouping of different germ cell tumors based on their associations with different treatments. On the other hand, the approach of using text mining to capture precision medicine cancer-related associations has its limitations. The most prominent is that associations are captured without any context, based solely on simple co-occurrence of terms of interest. This means that the type of association between cancer types, drugs or genes is not captured. Furthermore, the context of the association (e.g., different patient parameters, such as age, gender, ethnicity *etc.*) is ignored. The two examples given above also illustrate that there is some degree of noise in the data, such as in the association of chorionic gonadotropin to cancers that secrete this compound, rather than association to cancer types treated by chorionic gonadotropin. Nevertheless, we have demonstrated that even in its current simplistic form, the collection of information addressing therapy/gene/cancer type co-occurrences that we have assembled can be of use. Future improvement of this resource, through human editing and/or better text mining (see Section 2.3 *Future Directions*) can mold it into an even more valuable resource for cancer research.

### 2.3. Future Directions

As mentioned above, the PCM Wiki and Knowledgebase employs a straightforward approach to capturing patient case reports in precision cancer medicine. While the use of a Wiki does help overcome limitations of the case report submission form, the model of the form could be expanded to allow for capturing more complex cases (e.g., where more than one line of targeted treatment was used). Furthermore, by capturing the manner in which precision cancer cases are reported by Wiki users, standards of reporting can be assessed. Since such standards would be derived from user contributions, they are likely not only to be best suited for capturing precision cancer medicine-related knowledge but also to appeal to the community that would eventually be using such information.

In addition to improving the manner in which case reports are captured in the knowledgebase, more complex associations between cancer types, drugs and genes could be captured from the literature. In their current form, the Wiki information cards capture information about cancer/drug/gene co-occurrences from publications. Using more advanced text mining techniques, the type of relationships and their context could also be captured and represented for users of the Wiki, for example through the use of interactive networks.

We strongly believe that the interfaces included as part of the knowledgebase (such as the Wiki, the case submission form and the query engine) are user-friendly, straightforward and will appeal to a wide range of users. However, a structured assessment of the interfaces provided by the knowledgebase (for example, using a focus group that would “play around” with the interfaces and point out any issues) would be of great benefit for the further development and improvement of the knowledgebase.

Contributions of PCM Wiki and Knowledgebase information is on a voluntary basis, meaning that physicians add cases as they see fit rather than including all possible cases found in hospital records. Our choice to base the PCM Wiki and Knowledgebase on physician-contributed cases means that only interesting precision cancer medicine cases are captured at the cost of partial and biased coverage of the case spectrum. One possible future direction for the knowledgebase would thus be to allow patients to add their own cases. This would, however, require the development of a submission mechanism that is oriented at non-medically-educated users, and would have to include careful safeguards and filters to prevent mistaken or fraudulent entries from degrading the value of the knowledgebase. Furthermore, a mechanism to flag contributors differently will also be needed so to allow filtering of cases based on whether they were contributed by physicians or patients.

## 3. Experimental Section

### 3.1. Gene, Drug and Disease Lists

The list of gene names and synonyms was downloaded from the HUGO Gene Nomenclature Committee (HGNC) database (June, 2013).

The list of disease names and synonyms was extracted from the Disease Ontology (DO) [25] and a subset of cancer types from the Disease Ontology was created (Supplementary File S1).

The list of drugs and therapies was compiled from RxNorm [17] which provides normalized names for clinical drugs and links to many of the drug vocabularies (and is included as part of the Unified Medical Language System [26]).

### 3.2. Wiki, Knowledgebase and Web Development

The PCM Wiki was built using the MediaWiki platform [27]. The case report submission mechanism was developed using the MediaWiki Bulk Page Creator [28].

All web applications were developed using PHP and HTML.

**Figure 6:**
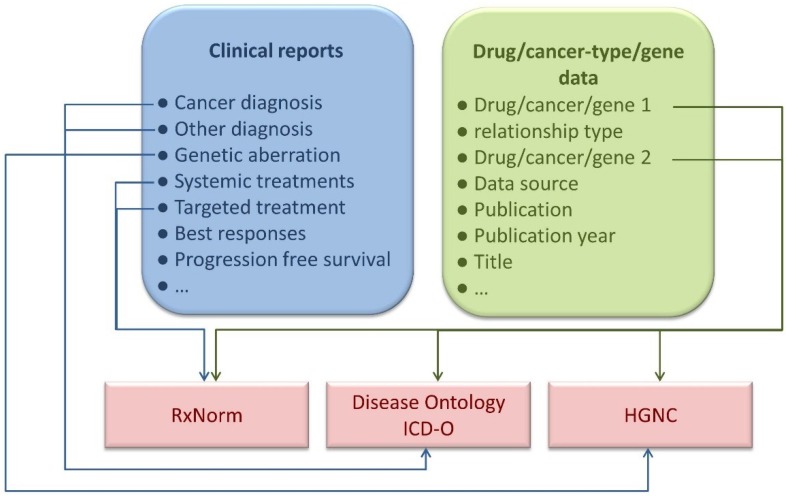
The PCM Database’s schema. The PCM database comprises two main data-tables: (1) A clinical reports table and (2) A drug/disease/genetics data table. In the clinical reports table, case reports and all pertaining information are stored. In the drug/disease genetics table, relationships between drugs, diseases and genetic markers (mined from various sources of information, such as PubMed abstracts) are stored in the form of triples. Each triple comprises a source entity (drug, disease or gene), a relationship type and a target entity.

The PCM database schema is described in Figure 6. In brief, the database comprises two tables:

(i) A clinical reports table containing description of each of the case reports in the knowledgebase.

(ii) A drug/disease/genetics data table containing information regarding relationships between drugs, diseases and genetic markers. The relationships are captured in the form of entity 1 (a drug, disease or gene) having a relationship of the type defined by “relationship type” with entity 2 (a drug, disease or gene).

### 3.3. Text Mining

In total, 2,115,767 PubMed abstracts were exported (October, 2012) from NCBI in the MEDLINE format, using the key word “human”. For each abstract, the list of associated MeSH [29] terms was recorded. The lists of gene names, drug and therapies and diseases (compiled as described above) were sought in the list of MeSH terms associated with each abstract.

For each abstract, the following information was captured and recorded: PubMed ID, publication year, title, publication, publication type, complete MeSH terms list, genes, drugs and diseases (matched from lists).

The mining script (implemented in Perl) is available as Supplementary File S2.

### 3.4. Clustering Analysis

A quantitative drug-disease matrix was generated by obtaining a count of the number of instances (abstracts) mapped to that disease and that drug for each cancer type and drug in the database. Similar matrices of drug-gene and gene-disease co-occurrences were also generated (Supplementary File S3).

Only drugs or therapies associated with the “pharmacology”, “Administration and dosage” and “Therapeutic use” MeSH categories were included. Cancer types were included only if they were associated with the “Diagnosis” category of MeSH and genes were included only if they were associated with the “Genetics” category of MeSH.

Using Expander 5 software [30], hierarchal clustering was conducted (with default parameters, using Pearson correlation as the similarity statistic). Complete clustering results are provided as Supplementary File S4.

### 3.5. Availability

The Precision Cancer Medicine Wiki and Knowledgebase is freely available for viewing and querying by all users. To make contributions and edits to the PCM Wiki, users must first register by sending a request to pcm@post.bgu.ac.il.

The Precision Cancer Medicine Wiki and Knowledgebase home page can be found at: http://rubinlab.bgu.ac.il/pcm/.

The Precision Cancer Medicine Wiki can be found at: http://pcm-wiki.bgu.ac.il.

The Precision Cancer Medicine Wiki case report submission form can be found at: http://pcm-crs.med.ad.bgu.ac.il/form.html.

## 4. Conclusions

We present here a novel and innovative approach and platform for capturing and presenting precision cancer medicine cases and related knowledge. The PCM Wiki and Knowledgebase is designed to become an important tool for the advancement of clinical trials and care in the field of precision cancer medicine.

## Supplementary Files

Supplementary File 1
